# Monkeypox: the resurgence of forgotten things

**DOI:** 10.4178/epih.e2022082

**Published:** 2022-09-26

**Authors:** Sun Bean Kim, Jaehun Jung, Kyong Ran Peck

**Affiliations:** 1 Division of Infectious Diseases, Department of Internal Medicine, Korea University College of Medicine, Seoul, Korea; 2 Artificial Intelligence and Big-Data Convergence Center, Gil Medical Center, Gachon University College of Medicine, Incheon, Korea; 3 Department of Preventive Medicine, Gachon University College of Medicine, Incheon, Korea; 4 Division of Infectious Diseases, Department of Medicine, Samsung Medical Center, Sungkyunkwan University School of Medicine, Seoul, Korea

**Keywords:** Monkeypox virus, Clinical features, Therapeutics, Vaccination

## Abstract

Monkeypox, a rare zoonotic disease, is primarily prevalent in Central and Western Africa. However, monkeypox is emerging as a worldwide concern due to the 2022 monkeypox outbreak, which is the first instance of widespread community transmission outside Africa. Monkeypox is caused by the monkeypox virus, which belongs to the genus *Orthopoxvirus* and presents as a vesicular-pustular disease that may be preceded by fever, malaise, and other constitutional symptoms. If present, lymphadenopathy may distinguish it from chickenpox or smallpox. However, contrary to previous manifestations, most monkeypox patients presented with atypical features during the 2022 outbreak. Monkeypox is usually a self-limiting disease with symptoms lasting between 2 weeks and 4 weeks and is mainly transmitted when a person comes into contact with an infected animal, person, or fomites contaminated with the virus. Very few treatment options are available for this disease. Tecovirimat has been licensed in some countries for the treatment of smallpox and monkeypox infections. Two other medications, cidofovir and brincidofovir, have been found to be effective against poxviruses in *in vitro* and animal studies, but data on human cases of monkeypox are limited. Although Imvamune (JYNNEOS), a vaccine against monkeypox, is authorized in the United States, there are currently no established routine vaccination programs. Current preventive strategies focus on the detection of probable cases and containment of the outbreak through the implementation of selected ring vaccination programs. Fundamental principles to prevent the spread of monkeypox, including maintaining personal hygiene and avoiding close contact with symptomatic patients, are of paramount importance.

## INTRODUCTION

Human monkeypox is caused by an *Orthopoxvirus* and constitutes an accidental zoonosis with symptoms comparable to those of smallpox, but less severe clinically [1]. Monkeypox still occurs in endemic countries in Central and Western Africa, particularly in the Democratic Republic of the Congo, despite smallpox being eradicated in 1980 [2]. Moreover, occurrences of monkeypox in Nigeria from 2017 to 2020 resulted from a loss in population-level immunity following the end of smallpox immunization in the 1980s [3]. Monkeypox can be distinguished from other rash illnesses using clues from patients’ histories, such as recent travel to endemic regions, contact with wild animals imported from endemic regions, and caring for an infected animal or person.

The 2022 monkeypox outbreak, which was first discovered in the United Kingdom in May 2022 and subsequently confirmed in at least 89 countries across 6 global regions, is the first instance of widespread community transmission outside Africa [4]. The World Health Organization (WHO) declared the increasing global monkeypox outbreak a public health emergency of international concern on July 23, 2022 [5]. As of August 7, 2022, the United States and some European countries had the highest total number of cases reported worldwide [4].

## HISTORY

Two outbreaks of a disease resembling smallpox occurred in colonies of monkeys sent from Singapore to Denmark for research in 1958, giving rise to the name “monkeypox” [6]. When smallpox elimination efforts were ramped up, the first human case of monkeypox was found in 1970 in a child from the Democratic Republic of the Congo [7]. Human monkeypox cases have primarily been discovered in Western Africa, but since 1981, the Congo Basin of Central Africa has seen most of the cases [8]. The majority of human monkeypox cases are still being reported from the Democratic Republic of the Congo [9]. Human monkeypox outside of Africa has been connected to international travel or the importation of animals, notably in the United States, Israel, Singapore, and the United Kingdom [10]. The United States had the first zoonotic outbreak of human monkeypox outside of Africa in the spring of 2003 [11].

## EPIDEMIOLOGY AND PATHOGENESIS

The monkeypox virus, a double-stranded DNA virus of the genus *orthopoxvirus*, family Poxviridae, is the culprit behind the uncommon zoonotic disease known as monkeypox [12]. The variola virus, which causes smallpox, the vaccinia virus, which is used in the smallpox vaccine, the cowpox virus, and the camelpox virus are all members of the *orthopoxvirus* genus [12]. Two separate phylogenetic subclades of the monkeypox virus were identified in the past: the Congo Basin (Central African; clade 1) and Western African (clade 2) [13]. These 2 clades demonstrate epidemiological and clinical distinctions and are geographically matched [14]. The Western African clade’s involvement in the 2003 United States pandemic led to the hypothesis that illness severity varies across clades [13]. Liberia, Sierra Leone, Nigeria, and Côte d’Ivoire were the only countries in Western Africa to report monkeypox cases, with each country reporting only 10 cases between 1970 and 2005 [8]. The majority of cases belonging to the Western African lineage, however, were associated with the outbreak that resulted in 37 cases in the United States in 2003 [15], and the outbreak in Nigeria that began in 2017 [16]. In addition, this clade has been discovered in cases involving travel from Israel [17] and Singapore [18]. In general, Western African monkeypox is milder in both humans and non-human primates, [13,19,20] whereas the disease caused by the Congo Basin clade has a reported case fatality rate of up to 11% [14].

Since January 1, 2022, cases of monkeypox have been identified in 89 countries, territories, and areas, spanning six WHO regions [4]. Since May 7, 2022, there have been more cases of monkeypox recorded outside of Africa than there were from 1970 to the present outbreak [21]. As of August 7, 2022, 27,814 laboratory-confirmed cases including 11 fatalities, had been reported to the WHO [4], the majority of which (98%) have been detected since May [4]. The Unite States (n= 7,510), Spain (n= 4,577), Germany (n= 2,887), United Kingdom (n= 2,759), France (n= 2,239), Brazil (n= 1,721), the Netherlands (n= 959), Canada (n= 957), Portugal (n= 710), and Italy (n= 505) are the 10 countries with the highest total number of cases reported worldwide as of August 7, 2022, and 89% of the cases documented internationally to date are from these countries [4]. The 2022 monkeypox virus belongs to the new West African clade 3; based on preliminary genetic data, this clade is most closely related to the monkeypox viruses associated with the disease’s transmission from Nigeria to the United Kingdom, Israel, and Singapore in 2018 and 2019, and then from a 2021 travel-associated case from Nigeria to the United States [16,22-26]. According to a recent Centers for Disease Control and Prevention (CDC) analysis, new genetic sequencing data show that at least 2 separate outbreaks of monkeypox are occurring outside of Africa [27]. Three of the 10 viruses from recent United States cases of monkeypox that the CDC has sequenced - 2 from 2021 and 8 from 2022 - are distinct from the viruses sequenced by many other countries implicated in the large outbreak that is moving throughout, and from, Europe [27]. Despite having a similar ancestor and being plainly related to one another, the 3 divergent viruses are very different from one another and other viruses [27]. The virus infected these 3 patients from 3 different geographic locations: 1 in Nigeria, 1 in another Western African country, and 1 in either the Middle East or Eastern Africa [27]. This apparent widespread dissemination of a closely related virus - one that is distinct from the strain that triggered the European outbreak - suggests the occurrence of monkeypox outbreaks outside of the countries where the disease has been more widely spread than anticipated [27].

For 73% (17,052/23,290) of cases, information about gender is available; the majority of infected individuals (16,839/17,052) were men, with a median age of 36 years (interquartile range, 31-43). Since men between the ages of 18 years and 44 years account for 77% of reported cases, they continue to be disproportionately affected by this outbreak. Fewer than 1% (98/17,426) of the cases with age information were 0-17 years old [4]. Of the cases with known human immunodeficiency virus (HIV) status, 39% (3,204/8,234) were HIV-positive [4]. There have been 344 cases reported to date involving healthcare personnel; in the current outbreak, at least 1 occupational exposure has been reported, despite the majority of cases being contracted in the community [4]. With the exception of countries in the African region, men who identify as gay, bisexual, or men who have sex with men and who have reported recent intercourse with 1 or more partners continue to be the major groups affected by the ongoing monkeypox outbreak [4]. Despite the fact that cases have also been documented in women, children, and other groups of men, there is little evidence to suggest that these new groups are experiencing sustained transmission [4]. Moreover, given the initial lack of epidemiological connections to endemic locations, the sudden emergence of monkeypox in various areas raises the possibility of long-term, undetected transmission [28].

## ORIGIN AND SPREAD OF MONKEYPOX VIRUS

Numerous poxviruses are named after the animal host in which they were first discovered (such as cowpox and monkeypox), but the main reservoirs for these viruses may be rodents or other species such as African dormice, giant-pouched rats, and rope and sun squirrels in Western and Central Africa; for most, their primary animal reservoir remains unclear [29,30]. Further research is required to determine the specific reservoirs, as well as how virus circulation is maintained in nature [31]. The monkeypox virus has been shown to infect a variety of animal species, and non-human primates (such as monkeys) and African rodents may host the virus [31,32].

When a person comes into contact with an infected animal, person, or contaminated fomites, the monkeypox virus can spread [9,33]. The virus can potentially pass from a mother to a fetus through the placenta [34]. Additionally, people can contract the monkeypox virus from other animals by being bitten or scratched by an infected animal, handling wild materials, or using products derived from infected animals [33]. The virus can also be transmitted by direct contact with body fluids or lesions from an infected person, or contaminated fomites, such as clothing or linens [9,35]. Additionally, it can spread by respiratory secretions during close, prolonged contact, including face-to-face contact [35]. Monkeypox can be transmitted between people when they have sex, kiss, cuddle, or touch monkeypox lesions [10]. Although the monkeypox virus has never been identified as a sexually transmitted infection, the majority of recent case clusters involve men between the ages of 20 and 50, many of whom have had sex with other men [2]. As a result, it is thought that close contact during sexual activity is spreading the monkeypox virus [2]. A recent study found that seminal fluid samples were positive for monkeypox virus DNA in real-time polymerase chain reaction (PCR) [36]. The potential that the virus might be transmitted sexually is currently being investigated by the WHO [28]. It is currently unknown whether semen or vaginal fluids can spread monkeypox [28,37].

## CLINICAL FEATURES

Monkeypox incubation is typically 7-14 days, although it can range from 5-21 days [38]. The majority of the clinical characteristics of human monkeypox are similar to those of distinct, ordinary smallpox; however, they manifest milder symptoms (Table 1) [36,38,39]. Skin eruption and invasion are 2 pathways for infection. Fever, chills, severe headaches, back pain, fatigue, and myalgia are early signs of monkeypox. As a result, it is often initially diagnosed as influenza [1]. In many cases, a rash appears before, or concurrently with, the development of the aforementioned symptoms, and the submaxillary, cervical, or inguinal lymph nodes frequently expand (1-4 cm in diameter). The swollen lymph nodes are firm, tender, and occasionally painful [1]. The primary distinction between the signs and symptoms of monkeypox and smallpox is that the former usually results in lymphadenopathy while the latter does not [1,40]. According to one theory, the prevalence of lymphadenopathy in monkeypox may indicate that the human immune system recognizes and responds to the monkeypox viral infection more effectively than it does the variola virus infection [19]. A patient experiences fever onset within 1 day to 3 days (rarely longer), followed by the appearance of a rash. In 95% of cases, the rash starts on the face and then quickly spreads to other areas of the body in a centrifugally concentrated pattern, including the palms of the hands and soles of the feet (in 75% of cases) [41]. Lesions often proceed to the same stage in all body parts [41,42]. The most commonly affected areas are the oral mucous membranes (in 70% of cases), genitalia (30%), conjunctivae (20%), and cornea [41]. The distinctive lesions begin as macules before developing into papules, vesicles, and pustules [41,43]. Oral lesions, which are frequently present, might cause swallowing difficulties [37]. Swelling, stiffness, and pain ensue as a result of the skin’s significant disruption, and eventually crusts form [6]. In comparison to those who are not immunized, vaccinated patients were said to have fewer, smaller lesions, better presentation of regional monomorphism, and centrifugal distribution of rash [38]. Secondary bacterial skin infections were found in 19% of non-immunized patients with monkeypox [6,7]. The deterioration of the patient’s condition as a whole has been linked to a second febrile phase that starts when skin lesions turn pustular [6]. Patients with HIV may experience different symptoms [44]. Since June 1 of this year, 66 people have already died from monkeypox in African countries; however, most recent cases recorded in non-endemic countries have been rather mild [45]. Additionally, a large number of patients in the 2022 monkeypox outbreak displayed unusual characteristics, such as a few lesions or a single lesion, lesions that started in the genital or perineal/perianal area and did not spread to other parts of the body, lesions that appeared at various (asynchronous) stages of development, and lesions that appeared before the onset of fever, malaise, and other constitutional symptoms [10,28,39,40,46]. Typically, monkeypox is a self-limiting illness with symptoms lasting between 2 weeks and 4 weeks. Based on the original Congo Basin cohort from 1981 to 1986, the convalescent periods were similar for unvaccinated smallpox patients (70% recovered after 3 weeks) and previously vaccinated smallpox patients (81% recovered after 3 weeks) [6]. The interval between receiving a smallpox vaccination and monkeypox presentation ranged from 3 years to 19 years [7]. Young age, the extent of virus exposure, prior immunization, nutritional status, and the presence of underlying immunologic or concurrent diseases are all associated with severity [6]. In addition to bacterial superinfection of the skin, irreversible skin scarring, hyperpigmentation or hypopigmentation, bronchopneumonia, sepsis, encephalopathy, and persistent corneal scarring with subsequent vision loss are sequelae of monkeypox [1]. Children and young adults have a higher mortality rate, and immunocompromised individuals tend to have a more severe disease course [15,47]. While multi-organ failure, hypotension, and coagulopathy are the primary causes of death from smallpox [48], sepsis due to secondary bacterial infection, encephalitis, and concomitant opportunistic infections are known to be possible causes of death from monkeypox [19,49]. It is unclear to what degree asymptomatic infections may develop [10]. During the outbreaks in the Congo Basin, case fatality rates ranging from 1% to 10% were recorded [50,51], and the viral clade circulating in this area showed greater virulence [13]. The Western African clade, however, which is responsible for the current outbreaks in Nigeria, has a lower overall death rate (3%) [14,16,51]. Other rash conditions included in the differential diagnosis are chickenpox, smallpox, disseminated zoster, disseminated herpes simplex, generalized vaccinia, measles, eczema herpeticum, molluscum contagiosum, rickettsialpox, bacterial skin infections, scabies, syphilis, and gonorrhea [10,46].

### Diagnosis

Laboratory tests are necessary to establish the diagnosis because the monkeypox virus is clinically difficult to distinguish from other pox-like illnesses [52]. Because of its high accuracy and sensitivity in determining the presence of *orthopoxvirus* or monkeypox virus in roof or fluid samples from vesicles and pustules, as well as dry crusts, PCR is preferred [53,54]. A biopsy is an alternate possibility in cases where it is practical [10]. Due to the short length of viremia compared to the timing of specimen collection following the onset of symptoms, PCR blood test results are typically equivocal; as a result, samples should not be routinely taken from patients [10,52,55]. Antigen and antibody detection tests do not offer proof of monkeypox-specific infection because *orthopoxviruses* are serologically cross-reactive [10,52,55]. If there are insufficient resources, serology and antigen detection procedures are not advised for diagnosis or case evaluation [55]. Furthermore, recent or remote immunization with a vaccinia-based vaccine (for example, anyone immunized prior to the eradication of smallpox or more recently due to higher risk, such as *orthopoxvirus* laboratory employees) may result in false-positive results [10,52,55]. Conventional tests such as immunohistochemistry, electron microscopy, and virus isolation from clinical specimens are still valid methods, but they require complex laboratory equipment and highly skilled technicians [55]. Virus isolation should only be performed by personnel vaccinated against smallpox; furthermore, viral isolation should only be undertaken in laboratories with Biosafety Level-3 containment facilities and following validated protocols [56,57].

### Treatment

Many monkeypox virus patients experience a mild, self-limiting disease course without specific therapy [58]. The prognosis of monkeypox, however, is based on several variables, such as prior vaccination history, co-occurring diseases (such as malaria, varicella, and HIV), and underlying comorbidities [59]. Immunocompromised patients [58,60], people under the age of 8 years [7], and pregnant or breastfeeding women may be at a higher risk of developing a serious illness [61]. Antiviral therapy should be considered for patients with one or more complications [62].

No specific therapies are currently approved for monkeypox [23]. Like other viral illnesses, monkeypox is mainly treated conservatively [63]. Antivirals used for smallpox patients may be effective [64]. However, there is little definitive data to support this finding. Since smallpox was declared eliminated in 1980, no agent has been tested for its effectiveness in humans; nevertheless, in animal models, the compounds listed in Table 2 have shown efficiency against other *orthopoxviruses*, including monkeypox [65-70].

The antiviral intracellular viral release inhibitor tecovirimat, marketed under the trade name TPOXX, has received European Commission approval for the treatment of various diseases, including monkeypox, smallpox, cowpox, and vaccinia [58]. After being challenged with a number of *orthopoxviruses*, tecovirimat treatment increased survival in rats compared to placebo treatment, and this effect was seen even when the drug was given at a late stage of the disease [68,71-73]. In July 2018, the U.S. Food and Drug Administration (FDA) approved it for use in treating smallpox in adults and children who weigh at least 13 kg to mitigate the effects of a potential outbreak or bioterrorist assault [74]. For the treatment of non-variola *orthopoxviruses* (such as monkeypox) during an outbreak, the CDC adopted an expanded-access investigational new drug procedure (EA-IND) [7]. With no verified safety signals, some previous reports described the compassionate use of tecovirimat as a therapy for complex vaccinia [75,76] and cowpox [77].

In 2021, a patient receiving tecovirimat (600 mg twice daily for 2 weeks orally) had a shorter length of viral shedding and illness (10 days of hospitalization) than 6 other patients, according to a prior retrospective monkeypox study in the United Kingdom [64]. After receiving tecovirimat versus a placebo, macaques with smallpox were shown to have fewer lesions and a shorter duration of PCR positivity in the blood and upper respiratory tract [67]. Headache, nausea, and abdominal pain are the most frequently reported side effects. Approximately 360 human volunteers received tecovirimat as part of an expanded safety trial, which revealed the occurrence of side effects comparable to that of a placebo [71].

The use of tecovirimat as post-exposure prophylaxis has not been authorized, but an application to extend the license for this indication is underway [64]. In the Central African Republic, where outbreaks of monkeypox are prevalent, an expanded-access program for post-exposure tecovirimat prophylaxis was started in March 2022 [78]. Only the U.S. Government’s Strategic National Stockpile (SNS) has access to this agent [58]. In June 2021, the FDA also authorized brincidofovir, marketed as Tembexa, as a smallpox therapy [79]. It is a cidofovir analog with improved oral bioavailability [79]. Brincidofovir may be safer than cidofovir [79].

In prairie dogs with established monkeypox virus infection, brincidofovir inhibited viral DNA polymerase, stopped DNA replication, and provided a modest survival benefit (29 vs. 14%), as well as a decrease in end-organ viral titers [80]. In 2018, brincidofovir was chosen for use in patients in the United Kingdom because it was widely accessible and could be accessed urgently by repurposing an existing supply from a local clinical trial [64]. Three individuals with monkeypox received brincidofovir, but the medication was discontinued after the patients’ liver enzyme levels were altered (a recognized side effect) [64]. Furthermore, it is challenging to understand the connection between brincidofovir and the disease course given the limited number of samples used [64]. To simplify the use of brincidofovir for monkeypox treatment, the CDC is currently creating an EA-IND [58]. However, brincidofovir is not currently accessible via SNS [58].

The FDA has approved the antiviral drug cidofovir (also known as Vistide) for the treatment of cytomegalovirus retinitis in people with acquired immune deficiency syndrome [81]. Cidofovir is efficacious against lethal monkeypox challenges in animal models and possesses anti-monkeypox *in vitro* activity [59,82,83]. However, there are no clinical studies supporting its effectiveness in preventing monkeypox virus infection in humans, and it has the potential to cause serious side effects, such as nephrotoxicity [58]. The CDC put in place an EA-IND permitting the use of cidofovir for the treatment of monkeypox and other *orthopoxvirus* infections during an outbreak [58].

The FDA has approved the use of vaccinia immune globulin intravenous (VIGIV) for treating post-vaccination complications, such as eczema vaccinatum, progressive vaccinia, severe generalized vaccinia, vaccinia infections in people with skin conditions, and aberrant infections caused by the vaccinia virus, with the exception of patients with isolated keratitis. During the outbreak, the CDC devised an EA-IND to use VIGIV to treat infections caused by *orthopoxviruses*, including monkeypox [58].

### Prevention

Avoiding contact with animals that potentially harbor the virus, such as ill or dead animals discovered in areas where monkeypox occurs, is a strategy that has been used to prevent monkeypox virus infection [17,58]. Avoiding contact with sick animals, including bedding; isolating infected patients from those who might be at risk for infection [11,41,82,84]; practicing good hand hygiene after contact with infected people or animals [33] (such as washing hands with soap and water or using an alcohol-based hand sanitizer); using personal protective equipment when providing patient care [53]; and educating people about risk factors and preventive steps to reduce virus exposure are methods to limit infections.

Scientific research is now being conducted to determine the feasibility and appropriateness of vaccination for the prevention and control of monkeypox [41,85]. Some countries have policies in place or are creating them to provide vaccines to people who may be at risk, including laboratory staff, members of rapid response teams, and healthcare professionals [41,58].

### Vaccination

Although smallpox immunization provides some protection, the halt of smallpox vaccination campaigns worldwide after the elimination of the disease may have made persons between the ages of 40 and 50 more susceptible to monkeypox [41]. A selective approach, such as ring vaccination of confirmed cases, or mass vaccination as soon as the first case is diagnosed are 2 strategies that can be implemented if an outbreak begins [86]. In order to successfully control the disease and mitigate further spread, ring vaccination is defined as the prompt immunization of a person who has been identified as having been exposed to an infected person [86]. It has already been used successfully to contain Ebola outbreaks and could work against monkeypox [86,87]. Monkeypox is unlikely to become a pandemic according to the CDC and WHO [27,41]. Currently, the threat to the general public is relatively low [27,45]. Therefore, it is essential to detect possible cases and contain any outbreaks [86].

Based on the WHO’s interim recommendations for vaccines and immunization against monkeypox, mass vaccination is not recommended for monkeypox outbreaks, and vaccination is not currently recommended for the general population because of the relatively low risk to the public (Table 3) [88]. When available, pre-exposure vaccination and post-exposure prophylaxis are recommended for people who have had close exposure to a patient with monkeypox [88]. Tables 4 and 5 describe the interim recommendations made by the WHO regarding the use of vaccines for monkeypox pre-exposure and post-exposure prophylaxis based on exposure risk [88].

To prevent the spread of the virus, countries like Canada, the United Kingdom, and the United States have begun implementing a strategy called ring vaccination [63]. As a result of the viruses spreading to persons who have come into contact with an infected patient, smallpox vaccines are being administered, which are believed to be effective against monkeypox [63]. Ring vaccination can be an effective strategy; however, to be powerful, it must be implemented early in the course of an outbreak, while the number of infections is still under control [63].

Since June 2, 2022, the Korea Disease Control and Prevention Agency has hosted sessions of the Vaccination Expert Advisory group (VEAG) on the topic of vaccination against monkeypox. The VEAG advised pre-exposure and post-exposure prophylaxis for the high-risk target population, and the risk target group and recommendations for monkeypox vaccination in Korea were determined based on the probability of exposure (Tables 6 and 7) [89].

#### Smallpox immunization

According to a number of observational studies, the smallpox vaccine has an 85% success rate in preventing monkeypox [1,14]. Therefore, prior smallpox immunization may result in milder sickness [90,91].

In 1979—1 year before the WHO declared smallpox eradicable—smallpox immunization was stopped in Korea [92]. The firstgeneration (original) smallpox vaccines are currently no longer accessible to the general public [41,58]. To combat smallpox and monkeypox, a more recent vaccine based on the modified attenuated vaccinia Ankara vaccine (marketed as Imvamune and JYNNEOS) was authorized in September 2019 [93]. This is a 2-dose vaccine that is rarely available in the market [58]. Due to the cross-protection brought about by the immune response to *orthopoxviruses*, smallpox and monkeypox vaccines have been formulated utilizing the vaccinia virus [1,41,58].

The Advisory Committee on Immunization Practices (ACIP) recommended the use of this vaccine in 2021 for a group of workers who were at high risk of exposure to *orthopoxviruses*, such as those working in research laboratories, specialized clinical laboratories performing *orthopoxvirus* diagnostic testing, and designated response personnel who might be exposed to *orthopoxviruses* at work [58,94].

The ACAM2000 vaccine, a live, replication-competent vaccine for active immunization against smallpox, and the management of patients infected with replication-competent *orthopoxviruses*, is recommended by the ACIP for healthcare professionals [94]. The new ACIP smallpox vaccine guidelines were approved by the CDC in 2022 [84]. It is recommended that, after completing the primary JYNNEOS series, persons who are at continued risk of occupational exposure to more virulent *orthopoxviruses*, such as variola virus or monkeypox virus, receive booster doses of JYNNEOS every 2 years [94]. It is also recommended that following the primary JYNNEOS series, persons who continue to be at risk of occupational exposure to replication-competent *orthopoxviruses*, such as vaccinia virus or cowpox virus, receive booster doses of JYNNEOS at least every 10 years [94].

People who had an ACAM2000 primary immunization and were still at risk for occupational exposure to *orthopoxviruses* were recommended to receive a JYNNEOS booster instead of the ACAM2000 vaccine [84]. For people at risk of occupational exposure, the CDC Drug Service offers 2 *orthopoxvirus* vaccinations [95]. The U.S. FDA has approved JYNNEOS, a live, non-replicating vaccine, for use in persons 18 years of age and older who are determined to be at high risk of contracting smallpox or monkeypox [94], and ACAM2000, a live vaccine for active immunization against smallpox, for use in people at a high risk of contracting the disease [58,94].

A study on person-to-person transmission of monkeypox in Africa proved the value of smallpox immunization with vaccinia virus [85]. In this study, 2,278 household contacts’ secondary attack rates varied significantly according to their level of smallpox immunization (7.5% compared with the 1.3% rate in unvaccinated and vaccinated individuals, respectively) [85]. Vaccinated people had a 5-fold lower risk of monkeypox than unvaccinated people (0.78 vs. 4.05 per 10,000), according to another study [96] that revealed an increase in the number of human monkeypox cases in Africa. The vaccine’s effectiveness was estimated to be around 81% in those with a distant history of smallpox vaccination [96]. Additional research on the 2003 United States outbreak using experimental methods identified 3 additional patients who had been exposed to the monkeypox virus and had previously had smallpox vaccinations 13 years, 29 years, and 48 years prior [91]. These people did not exhibit any observable symptoms; therefore, they were unaware that they were infected [91]. These data imply that the monkeypox outbreak in the United States was more widespread than previously thought [91]. Furthermore, decades after receiving a smallpox vaccination, cross-protective antiviral immunity against West African monkeypox may be maintained [91].

### Post-exposure prophylaxis

The CDC states that vaccination can be considered up to 14 days after close contact exposure based on previous data on post-exposure vaccination for smallpox with the vaccinia vaccine. The ideal time for monkeypox post-exposure vaccination is 4 days [97]. Close contact is defined as direct exposure within 6 feet of an animal with a probable or confirmed case of monkeypox and displaying respiratory symptoms such as nasal discharge, coughing, or conjunctivitis [97].

The CDC recommended vaccination with vaccinia virus for a limited number of people exposed to monkeypox during the United States outbreak of 2003, including children and pregnant women, because prior vaccination with the vaccinia virus provided protection against monkeypox [95]. For this purpose, 28 adults and 2 children received the smallpox vaccine; none of these recipients contracted monkeypox [95]. Furthermore, during the 2003 outbreak, none of the pregnant women contracted monkeypox; therefore, it is uncertain whether pregnant women with this illness would have had a different outcome [98].

### Pre-exposure prophylaxis

Pre-exposure prophylaxis is recommended for healthcare workers at high risk of exposure, researchers manipulating *orthopoxviruses*, researchers working in clinical laboratories diagnosing monkeypox, and responders to outbreaks, as designated by national public health authorities [88]. Pre-exposure vaccination was also recommended by the CDC for people working on the outbreak investigation, as well as for medical personnel treating patients with monkeypox [95].

## VACCINATION FOR SPECIAL POPULATION GROUPS

If a vaccine suitable for these populations is available, vaccination against monkeypox as post-exposure prophylaxis may be considered for specific demographic groups, such as pregnant women, children, or people with immune suppression, including those living with HIV [88]. In general, pre-exposure vaccination against monkeypox is not recommended for these groups [88]. Clinical considerations, however, have been recently developed by the CDC for the prevention and treatment of monkeypox in people living with HIV; these include pre-exposure and post-exposure prophylaxis with the JYNNEOS vaccine [99]. The selection and timing of vaccination for people who might be exposed must be based on a thorough risk-benefit analysis and shared clinical decision-making reflecting the person’s individual circumstances [88].

## PERSPECTIVES

Smallpox vaccination was discontinued in 1980, resulting in a decrease in immunity against *orthopoxviruses*. Eradication of smallpox and waning immunity after vaccine cessation could be associated with the recent increase in monkeypox cases. In line with the increasing number of patients infected with monkeypox virus worldwide, a patient infected with monkeypox was first reported in June 2022 in Korea [100]. To prevent the spread of infectious diseases, it is essential to strengthen surveillance and establish an epidemiological investigation system, diagnostic tools, and strategies for contact tracing and isolation. With the experience in combating coronavirus disease 2019, the system for managing contagious diseases such as monkeypox is much more advanced than it was a few years ago. Although the clinical features of monkeypox, which is currently spreading, are mild, therapeutics and vaccines should be used appropriately to manage monkeypox outbreaks. Currently, Korea has stockpiled enough smallpox vaccine (second generation) to inoculate the entire population as preparedness for bioterrorism, and as a third-generation vaccine (JYNEOS) is safer and more convenient to inoculate, this vaccine should be prepared for preventive strategies against monkeypox transmission [89].

The focus is on detecting probable cases and containing the outbreak as quickly as possible through ring vaccination among the high-risk groups. Not only vaccinations but also fundamental principles to prevent the spread of monkeypox, including handwashing, personal hygiene, and avoiding close contact with symptomatic individuals, are of paramount importance. In addition, active self-reporting and testing are required if someone has a travel history abroad and contact history with infected patients or those with suspicious symptoms. This global crisis can be overcome through participation and solidarity.

## Figures and Tables

**Table 1.** Differences in clinical features between monkeypox, smallpox, and varicella infections

Characteristics		Monkeypox (typical) [38]	Monkeypox (atypical current outbreak) [36,37,39]	Smallpox [38]	Varicella [38]
Time period (day)					
	Incubation period	7-17	7-17	7-17	10-21
	Prodromal period	1-4	1-4	1-4	0-2
	Rash period	14-28	14-28	14-28	10-21
Symptoms					
	Prodromal fever	Yes	Occasionally	Yes	Uncommon, mild fever if present
	Fever (°C)	Yes, often between 38.5 and 40.5	Occasionally	Yes, often > 40.0	Yes, up to 38.8
	Malaise	Yes	Occasionally	Yes	Yes
	Headache	Yes	Occasionally	Yes	Yes
	Lymphadenopathy	Yes	Occasionally	No	No
	Lesions on palms or soles	Yes	Occasionally	Yes	Rare
	Lesion distribution	Centrifugal	Centrifugal	Centrifugal	Centripetal
	Lesion appearance	Hard and deep, well-circumscribed, umbilicated	- Raised, firm margins in the perianal and penile area	Hard and deep, well-circumscribed, umbilicated	Superficial, irregular borders, dew drop on a rose petal
			- Filled with clear fluid, and had surrounding erythema on the trunk, arm and leg		
	Lesion progression	Lesions are often in 1 stage of development on the body; Slow progression with each stage lasting 1-2 day	Only a few lesions or even just a single lesion; Lesions that begin in the genital or perineal/perianal area and do not spread further; Lesions appearing at different stages of development	Lesions are often in 1 stage of development on the body; Slow progression with each stage lasting 1-2 day	Lesions are often in multiple stages of development on the body; Fast progression

**Table 2.** Therapeutic options for the treatment of *orthopoxvirus* infections [38]

Antiviral therapeutic	Mechanism of action	Clinical considerations	Approval
Tecovirimat	Inhibits release of intracellular virus	Available oral and intravenous administration	- Licensed for the use of human smallpox disease in adults and pediatric patients weighing at least 13 kg in Jul 2018 by FDA
			- Licensed for the use of *Poxviridae* infections, including smallpox, monkeypox, cowpox, vaccinia in adults and pediatric patients weighing at least 13 kg in Jan 2022 by EMA
Brincidofovir	Modified cidofovir compound; Inhibits DNA polymerase	Lacks nephrotoxicity seen with cidofovir; Oral administration	- Licensed for the use of human smallpox disease in adults and pediatric including neonates in Jun 2021 by FDA
Cidofovir	Inhibits DNA polymerase	Intravenous administration with hydration and probenecid; Nephrotoxicity has been seen	- Licensed for the use of cytomegalovirus retinitis in AIDS patients in Jun 1996 by FDA
			- Has been used to treat other poxvirus infections (molluscum contagiosum and Orf virus)

FDA, U.S. Food and Drug Administration; EMA, European Medicines Agency; AIDS, acquired immune deficiency syndrome.

**Table 3.** Smallpox and monkeypox vaccine options [88]

Vaccine (manufacturer)	Licensed for smallpox (country, type, date)	Licensed for monkeypox (country, type, date)	Considerations	Advantages	Disadvantages
Modified vaccinia Ankara by Bavarian Nordic (MVA-BN), third generation	- EU: Imvanex has been authorised under exceptional circumstances (2013)	- US: Full MA (2019)	Very limited supply Liquid-frozen formulation, approved for use in the general adult population	The virus has limited replication in mammalian cells; No lesion produced at the vaccination site	Two-dose administration by injection
	- Canada: Full MA (2013)	- Canada: Full MA (2019)			
	- US: Full MA (2019)				
LC16 (KM Biologics), third generation	- Japan: Full MA (1975)	No	Approved for use in infants and children as well as adults (all ages)	Single-dose administration; Exhibits a safer profile and fewer adverse events than ACAM2000 in human and animal vaccinations	Attenuated virus that can still replicate in mammalian cells
	- US: EIND (2014)				
ACAM2000 (Emergent BioSolutions), second generation	- US approved	- US: EIND for PEP	Approved for use in adults aged 18-64 yr of age; Earlier production by Sanofi Pasteur approved in France	Single-dose administration; A successful take is noted by observation of a lesion at the vaccination site; Lyophilized preparation for long-term storage	Live viral vaccine that replicates in mammalian cells; Autoinoculation and contact transmission are risks; In low- disease-risk situations, should not be used for individuals with immunocompromising conditions, history of eczema or atopic dermatitis, or pregnant women; Cardiac events postvaccination have been noted to occur
Vaccinia, various strains^1^ from national production, first generation	Various countries various national production (SEP), held by various countries	No	Regular potency testing recommended	NA	No longer available to the general public

EU, European Union (European Medicines Agency); USA, United States of America (Food and Drug Administration); Canada, Health Canada; MA, market authorization; EIND, emergency investigational new drug program of the US Food and Drug Administration; PEP, post-exposure prophylaxis; SEP, smallpox eradication program; NA, not applicable.

1 For example, Wetvax/APSV; Lister/Elstree or Lancy-Vaxina.

**Table 4.** Use of vaccines for post-exposure prophylaxis against monkeypox according to exposure risk: WHO interim recommendations (June 2022) [88]

Exposure risk	Description of exposure		Post-exposure prophylaxis	Vaccine
High	Direct contact with the skin or mucous membranes of a person who has confirmed, probable, or suspected monkeypox, their body fluids (such as vesicular or pustular fluid from lesions), or possibly infected fomites (such as clothing or bedding), if the person is not wearing proper PPE; Inhalation of dust or droplets from cleaning contaminated rooms is an example of this		Post-exposure prophylaxis is recommended with a vaccine appropriate for each individual	ACAM2000, LC16 MVA-BN
		• Mucosal exposure from body fluid splashes		
		• Direct sexual contact with a person who has monkeypox or other contagious body touch; Face-to-face, skin-to-skin, mouth-to-skin, or exposure to body fluids or infected items or materials are all included in this (fomites)		
		• Regularly residing in the same house (regularly or occasionally) with a person who has been identified as having monkeypox during the period of alleged incubation,		
		or		
		• A sharp wound caused by a contaminated tool or by using infected gloves		
Medium	If not wearing proper PPE, avoiding direct contact but remaining in close proximity to a symptomatic monkeypox patient in the same room or indoor physical environment		Post-exposure prophylaxis is recommended with vaccine appropriate for each individual	ACAM2000, LC16 MVA-BN
Low/minimal	Contact with someone who has been diagnosed with monkeypox or with an environment that may be contaminated with the virus, while wearing proper PPE and without any known violations of the donning and doffing procedures or of the PPE itself		Post-exposure prophylaxis is not recommended	NA
		• Community contact or contact in an outdoor setting with a symptomatic case		
		• No known contact with a symptomatic case of monkeypox within the previous 21 day		
		or		
		• Personnel working in a laboratory handling routine clinical blood samples or other specimens not directly related to monkeypox diagnostic testing		

WHO, World Health Organization; PPE, personal protective equipment; NA, not applicable.

**Table 5.** Use of vaccines for pre-exposure prophylaxis against monkeypox according to the exposure risk: WHO interim recommendations (June 2022) [88]

Population group	Recommendations for vaccination (WHO SAGE, 2013)	Interim recommendations for vaccination (WHO Health Emergency Programme, 2022)
General population	Not recommended	Not recommended
Personnel working in research laboratory, clinical laboratory personnel conducting orthopoxvirus diagnostic tests, and designated response team members are all at risk of occupational exposure to monkeypox	Recommended	Recommended
	ACAM 2000, LC16 MVA-BN	ACAM 2000, LC16 MVA-BN
Above-mentioned: Individuals for whom standard replicating vaccine is contraindicated due to young age (children), pregnancy, immune deficiencies, immunosuppression therapies or atopic dermatitis	Recommended MVA-BN	Recommended LC16 MVA-BN

WHO, World Health Organization; SAGE, Strategic Advisory Group of Experts.

**Table 6.** An overview of recommendation and vaccination strategies for monkeypox in Korea [89]

Risk classification	Target population	Recommendation
Pre-exposure	Healthcare personnel in designated treatment team	Vaccinations are permitted with informed consent
	Laboratory personnel performing diagnostic testing for the monkeypox virus	
	Officers of health authorities (field epidemiologists, quarantine officers)	
Post-exposure	People exposed at a high level of risk within 4 day	Recommend upon informed consent
	People exposed at a high level of risk after 4-14 day	Vaccinations are permitted with informed consent
	People exposed at a medium level of risk within 4 day	
	People exposed at a medium level of risk after 4 day	NA
	People exposed at a low level of risk	

NA, not applicable.

**Table 7.** Stratification of exposure risk groups in Korea [89]

Exposure risk	Description of exposure
High	- Direct contact with symptomatic monkeypox cases through torn skin
	• Sexual or household close contact
Medium	- Only skin-to-skin contact; No torn skin
	• Clinical assessment of patients without wearing PPE
	- Passengers seated directly next to identified infected individuals
Low	- No direct contact
	- Direct contact with wearing appropriate PPE

PPE, personal protective equipment.
